# Development of nursing quality care process metrics and indicators for intellectual disability services: a literature review and modified Delphi consensus study

**DOI:** 10.1186/s12913-019-4749-y

**Published:** 2019-11-29

**Authors:** Owen Doody, Fiona Murphy, Rosemary Lyons, Anne Gallen, Judy Ryan, Johanna Downey, Duygu Sezgin

**Affiliations:** 1 Health Research Institute and Senior Lecturer, Faculty of Education and Health Sciences, Department of Nursing and Midwifery, University of Limerick, Castletroy, Limerick, Ireland; 2 Faculty of Education and Health Sciences, Department of Nursing and Midwifery, University of Limerick, Castletroy, Limerick, Ireland; 3 Lecturer, Faculty of Education and Health Sciences, Department of Nursing and Midwifery, University of Limerick, Castletroy, Limerick, Ireland; 4 Director of Nursing and Midwifery Planning Development Unit, National Lead for Nursing and Midwifery Quality Care Metrics Project, Health Services Executive Ireland North West, Bishop Street, Ballyshannon, Donegal Ireland; 5 Director of Nursing and Midwifery Planning Development Unit, Intellectual Disability Services Work-stream Chairperson, Health Services Executive Ireland South East, Kilkenny, Ireland; 6 Quality Care Metrics Project Officer, National Lead for Intellectual Disability Services Workstream, Health Services Executive, Ireland South, Cork, Ireland; 7 Postdoctoral Researcher, College of Medicine, Nursing & Health Sciences, Clinical Sciences Institute, National University of Ireland Galway, Costello Road, Galway, Ireland

**Keywords:** Consensus, Delphi study, Intellectual disability nursing, Nursing care process, Nursing metrics, Indicators, Quality care, Vulnerable population

## Abstract

**Background:**

Nursing process quality care metrics and indicators are quantifiable measures of the nursing care delivered to clients. They can be used to identify and support nurses’ contribution to high quality, safe, client care and are lacking in specialist intellectual disability nursing. In a national Nursing Quality Care-Metrics project for Irish intellectual disability services, a set of nursing quality care process metrics and associated indicators were established for intellectual disability services.

**Methods:**

A two-stage design approach was undertaken; a broad scoping review of the literature and a modified Delphi consensus process. The Delphi included a four round e-Delphi survey and a consensus meeting. Four hundred one intellectual disability nurses working in Ireland were recruited for the surveys and 20 stakeholders attended the consensus meeting.

**Results:**

From the review, 20 existing and 16 potential intellectual disability nursing metrics were identified for nurses to prioritise in the e-surveys. After the four survey rounds, 12 intellectual disability nursing metrics and 84 associated indicators were identified. Following the consensus meeting, these were reduced to 12 metrics and 79 indicators.

**Conclusions:**

This first set of intellectual disability nursing process metrics and associated indicators has been identified for implementation in practice. These metrics while developed in Ireland have international relevance and their application and appropriateness in practice needs to be evaluated.

## Background

Quality care and patient safety dominates research literature on healthcare [1] and is dependent on having dependable data and a structure to support analysis of such data. Poor nursing care and variations in nursing practices affect quality of care [2, 3] and generating indicators sensitive to nursing is difficult due to the invisible nature of nursing care [4, 5]. However, nursing metrics create the possibility to measure essential nursing care processes, recognise when care is dropping below the obligatory standard and enable staff to make enhancements to decrease the risk to patients\clients and their families [6]. This is relevant to people with intellectual disability (ID) as they are a vulnerable group as they experience; high levels of morbidity, hospitalisation, premature mortality [7] and health disparities [8]. While people with ID are living longer they are more likely to experience comorbidities [9, 10], with greater prevalence of coronary heart disease, cardiovascular disease, obesity and diabetes [11, 12].

Identifying measures of nursing care processes in specialist ID nursing (ID nursing) incorporate all actions related to care provision, from interpersonal relationships of care to technical delivery. This can be difficult to capture given the sometimes indiscernible nature of nursing and the fact that Ireland and the United Kingdom (UK) are the only countries with specific undergraduate programmes and registration for ID nurses. Additionally, there is limited evidence specific to ID nursing care metrics as compared to other branches of nursing. This may be compounded by the fact that not all nursing activities such as communication and compassion can be easily measured.

The concept of ID has evolved over the years to one where ID is now seen as a natural part of the human condition and defined by the environmental and social contexts in which the person lives [13]. ID nursing in Ireland originated in 1959 when it developed as a branch of nursing and initially was known as “Mental Sub-Normality Nursing”, and later changed to “Mental Handicap Nursing” before its present form “Intellectual Disability Nursing” [14]. In its early days, ID nursing services were delivered within institutional care settings employing an illness-oriented care model [14]. However, since the 1980s, a social model of care has been emphasised and ID services transferred from institutional care to community settings, focusing ID nursing on a biopsychosocial educational model [15]. Roles of ID nurses are multifaceted involving the delivery of person centred and holistic care. This includes the provision of health education, promotion and management, respecting individuals’ identity, providing multisensorial and alternative therapies, and supporting personal and social development [14]. ID nurses also support independent living, provide community based support and increase social awareness of people with ID and their needs [14, 16]. Within Ireland’s population (4.5 million), there are 28,388 people registered on the 2017 National Intellectual Disability Database (NIDD) receiving, or identified as needing, services for a diverse and complex range of health and social needs [17]. However the true total population of people with an ID in Ireland is undetermined [18] and internationally a 1 to 2% rate of the total population is generally accepted.

To value and make visible the role of ID nurses and improve client outcomes, there is a need to identify and measure current activities related to care processes. In Ireland a national research project was conducted to identify and develop a set of evidence-based nursing care process metrics and associated indicators across seven practice areas (acute care, mental health, public health nursing, children, midwifery, older person, and intellectual disability services). Each individual practice area group consisted of an academic team, a Nursing and Midwifery Practice Development (NMPD) Director (chair), the National Quality Care-Metrics lead, NMPD project officers, and key stakeholders including Directors of Nursing, clinical practitioners and service users. Within the project, the definition of quality care process metrics and indicators by Foulkes (2011) was adopted [19] (Table 1). Quality measures are frequently classified into three types; structure, process and outcomes [20]. Structure measures reflect factors such as the availability of staff and facilities. Process measures consider whether care interventions adhered to best practice guidance, and outcome measures consider the changes as a result of the care delivered. Outcome measures are frequently used in healthcare; however measuring process can be a more efficient measure of quality [21] as they are amenable to direct action to enable continuous improvement by care providers. While generally, metrics and indicators are a statement evaluated against an agreed standard, the fact the registration of ID nurses does not occur beyond Ireland and the UK fulfilling the aspect of evaluating against an agreed standard can be difficult. This emphasises the necessity for ID nursing to create it evidence base from and within practice, and this paper reports on the development of ID nursing process metrics and indicators identified through a scoping literature review and modified Delphi consensus process.

**Table 1:** Quality care process metrics and indicators [19]

Quality Care Process Metric	Quality Care Process Indicator
A quantifiable measure that captures quality in terms of how (or to what extent) nursing care is being performed in relation to an agreed standard.	A quantifiable measure that captures what nurses are doing to provide that care in relation to a specific tool or method.

## Methods

**Fig. 1:**
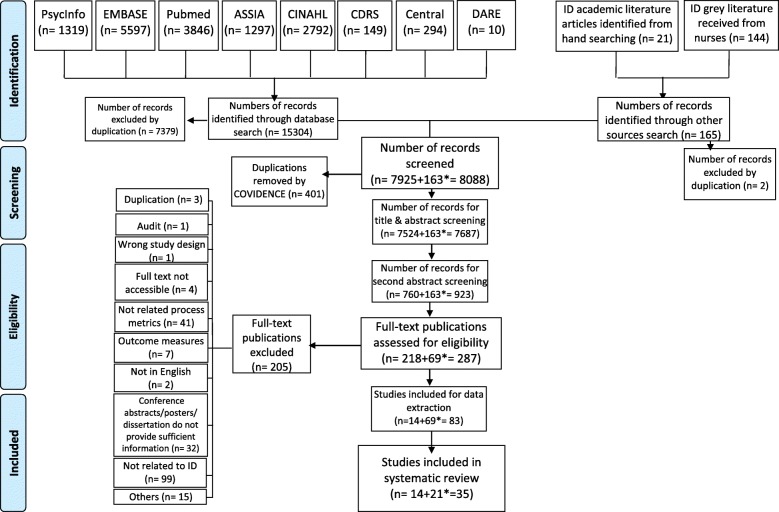
Study process

This study aimed to identify a set of process metrics and indicators for ID nursing. The study process (Fig. 1) consisted of two stages; a broad scoping review of the literature followed by a modified Delphi consensus process.

### Stage 1-broad scoping literature review

A broad scoping review of the literature using a systematic process using Moher et al’s [22] proven and robust processes and utilising Covidence online platform [23]. Scoping reviews are useful in areas where there may be a lack of empirical research evidence and conceptual ambiguity [24]. The search was not limited by study design but widened to include all types of sources, including grey literature. The literature search was originally conducted as a national collaboration across all seven practice areas (older persons services, intellectual disability nursing, mental health nursing, acute nursing, public health nursing, children’s nursing and midwifery). The aim of the review was to identify quality care process metrics and relevant indicators for nursing/midwifery, and to identify the current evidence base (last 10 years - 1st of January 2007 and 1st of January 2017) across eight databases (CINAHL, Psyinfo, EMBASE, ASSIA, Pubmed, CDRS, DARE, CENTRAL) and grey literature utilising the search terms: nurs*:ab,ti OR midwi*:ab,ti AND (‘minimum data set’:ab,ti OR indicator*:ab,ti OR metric*:ab,ti OR ‘quality measure*’:ab,ti) AND [english]/lim AND [2007–2017]/py.

**Fig. 2:**
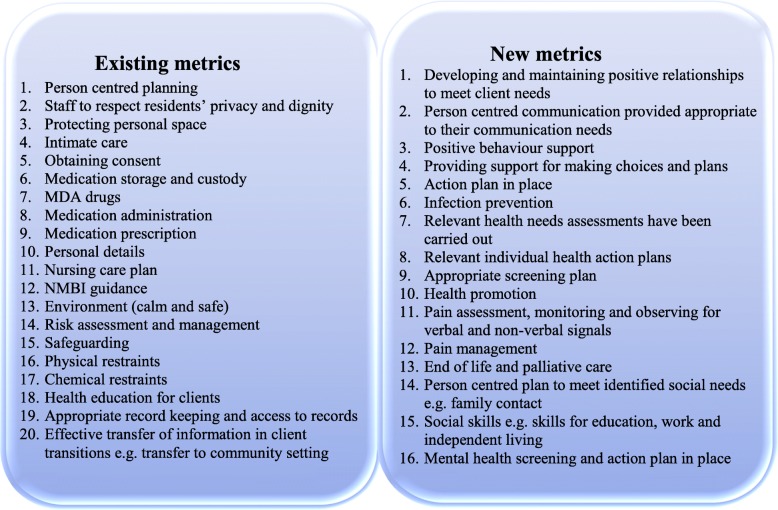
PRISMA Flow Diagram [24]

The database and grey literature search identified 15,304 results (Fig. 2), all titles and abstracts and full-text were reviewed independently by two reviewers and 112 publications met the inclusion criteria. No articles reported ID nursing process metrics and indicators. However, 14 articles were selected from the generic nursing literature to be used to extract ID nursing related process metrics and indicators. In addition, grey literature provided by ID services nationally were searched together with publications identified from hand searches. This resulted in a further 21 documents. In total, 35 publications were identified (Fig. 2) for review and data extraction and where appropriate, research studies were independently critically appraised using the Crowe Critical Appraisal Tool - CCAT [25].

### Stage 2: Delphi consensus process

A Delphi consensus process includes gathering expert opinions on an area to build consensus to reach an understanding of a problem with possible solutions using a series of questionnaires and group communication techniques [26]. A classical Delphi technique consists of the following features: anonymity, controlled feedback, iteration, statistical group response and stability [27]. In this study, a modified Delphi was used where the Delphi-consensus technique included a four round electronic survey of nurses and was modified by the addition of a face-to-face consensus meeting. Process metrics identified from the literature review were prepared for rounds one and two of the e-survey. Participants could vote on which metrics they considered important. In rounds three and four participants could vote on which indictors they considered important. This process concluded with a final face-to-face consensus meeting with key stakeholders to agree the final set of quality care process metrics and indicators for ID nursing. The consensus meeting of key stakeholders (*n* = 20) consideration grade and geographical representation and consisted of NMPD project officers, clinical practitioners (clinical nurse managers, clinical nurse specialists, staff nurses), practice development co-ordinators and service users representation.

For the Delphi surveys, a purposive sample was followed for recruitment of participants (nurses working in ID Services across Ireland). Therefore, ID nurses working in ID services nationally were recruited via national adverts and with the support of NMPD officers who distributed an information package to ID nurses (*n* = 4393). The sample size was calculated as 353 considering a representative of 4393 ID nurses, which would give a confidence level of 95% and confidence interval of ±5. Prospective participants had a chance to email the research team directly to obtain more information and clarify any issues before they made their decision to participate. Participants were emailed an invitation, further information with instructions related to the first e-Delphi survey instrument and the online survey link.

The end phase of the e-Delphi process comprised of a face-to-face consensus meeting with key stakeholders to review and discuss the findings from the e-Delphi surveys with the aim of building consensus on the relevance and wording of the final set of ID metrics and their associated indicators. The key stakeholders (*n* = 20) represented clinical expertise, nursing leadership and service provision across Ireland. The process was informed by a review of the literature carried out prior to the meeting, which identified ways to manage the consensus meeting [28–31]. To assist participants in voting for metrics and indicators, an evaluation tool was developed by the researchers following another review of the literature and expert review process.

#### Data collection

The e-Delphi surveys collected information related to demographics (work place, grade, year of experience) and included the list of metrics/indicators. Questions related to metrics/indicators asking ID nurses to rate each metric/indicator on a 9-item Likert scale (1 to 3 “not important”, 4 to 6 “important”, and 7 to 9 “very important”). Data collection occurred between June and October 2017 through online e-Delphi survey rounds with each round open for 21 days. All who expressed an interest to participate were provided with a weekly e-mail as a reminder/update. Experts participating in the face-to-face consensus meeting were asked to vote “yes/no” for each metric and indicator using a paper-based voting system.

### Data analysis

The findings of the scoping review informed the metrics in the survey rounds and the modified Delphi consensus technique was used to finalise the suite of ID nursing metrics and indicators through four rounds and a face-to-face consensus process. This enabled nurses working in ID services nationally to select metrics and indicators they deemed most relevant to their professional clinical practice. Data gained from the e-Delphi surveys was analysed using simple descriptive statistics to summarise data. Responses to each round were collated, analysed, and redistributed to participants for further comment in successive rounds and re-rated by participants, with knowledge of the group’s results from the previous round. Consensus on inclusion of a metric/indicator was agreed prior to commencement. It was determined that where 70% or more of nurses scored the metric/indicator as 7 to 9 and less than 15% scored the metric/indicator as 1 to 3 this metric/indicator was deemed included [32]. A similar approach was followed for the face-to-face meeting where metrics and indicators voted yes by more than 70% of the experts were included.

### Ethical issues

Ethical approval was obtained from the researchers universities Research Ethics Committee (No: 2016_12_12_EHS). Participants gave consent to participate by clicking on an ‘*I consent to participate in this study’* electronic link prior to being permitted to access the e-Delphi rounds. The online survey software system which maintained the survey data was protected behind a firewall. Only the research team had access to the data through use of a user identifier and password.

## Results

**Fig. 3:**
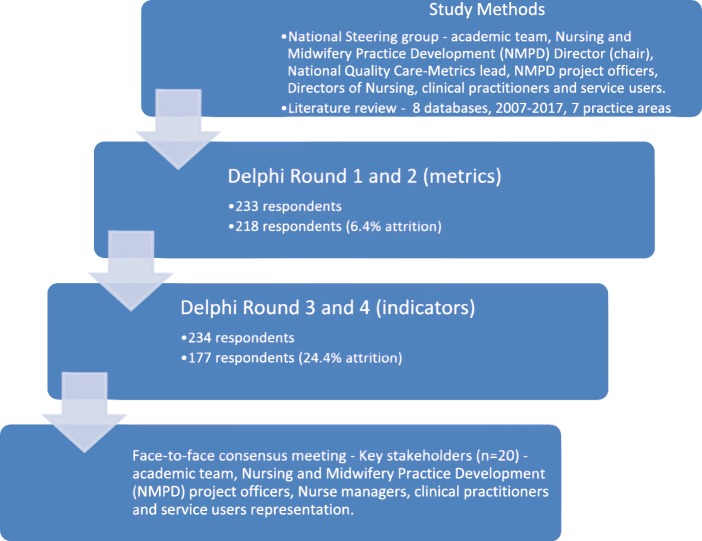
Existing and new ID metrics for Round 1 of the e-Delphi survey

Following the literature review process, 36 intellectual disability nursing metrics were identified. Sixteen new metrics were identified from the generic nursing literature and 20 current metrics from the 2015 practice areas Standard Operating Procedure for Nursing and Midwifery Quality Care-Metrics [33] (Fig. 3). Four hundred one nurses working in ID were recruited and an overall response rate of 50% was achieved for all rounds. Most of the nurses participating in all rounds were at staff nurse level and their average years of experience was over 20 (Table 2).

**Table 2:** Profile of the nurses participating in Delphi rounds

Characteristic	Round 1 *n* = 233 *n* (%)	Round 2 *n* = 218 *n* (%)	Round 3 *n* = 234 *n* (%)	Round 4 *n* = 177 *n* (%)
Grade				
Staff Nurse	72 (30.9)	56 (25.7)	68 (29.1)	56 (31.6)
CNM1^a^	29 (12.4)	35 (16.1)	29 (12.4)	16 (9.0)
CNM2^a^	47 (20.2)	48 (22.0)	43 (18.4)	28 (15.8)
CNM3^a^	14 (6.0)	15 (6.9)	16 (6.8)	10 (5.6)
Clinical Nurse Specialist	13 (5.6)	12 (5.5)	10 (4.3)	9 (5.1)
Director of Nursing	11 (4.7)	8 (3.7)	8 (3.4)	7 (4.0)
Assistant Director of Nursing	3 (1.3)	4 (1.8)	7 3.0)	6 (3.4)
Educator	4 (1.7)	5 (2.3)	5 (2.1)	1 (0.6)
Other	26 (11.2)	24 (11.0)	35 (15.0)	30 (17.0)
Not indicated	14 (6.0)	11 (5.0)	13 (5.5)	14 (7.9)
	*n* = 100^b^	*n* = 99^b^	*n* = 96^b^	*n* = 94^b^
Average years of experience	20.0	20.8	21.6	20.8
Range	1–40	1–40	1–40	1–40
	*n* = 151^b^	*n* = 130^b^	*n* = 152^b^	*n* = 108^b^
Health Service Regions	*n* (%)	*n* (%)	*n* (%)	*n* (%)
Region 1	48 (31.8)	42 (32.3)	48 (31.5)	32 (29.6)
Region 2	16 (10.6)	14 (10.8)	16 (10.5)	12 (11.1)
Region 3	35 (23.2)	30 (23.1)	35 (23.0)	21 (19.4)
Region 4	52 (34.4)	44 (33.8)	53 (35.0)	43 (39.9)

^a^*CNM* Clinical nurse manager (with levels 1, 2, and 3)

^b^Those who provided information on years of experience and region they worked in

A total of 233 ID nurses participated in round 1, resulting a response rate of 58.1%. Thirty-five of the 36 metrics were rated between 7 and 9 by 70% or more of the participants and rated between 1 and 3 by less than 15% of participants and hence included in round 2. In addition, participants had the chance to add suggestions for additional areas of practice (round 1) to be considered as possible metrics in round 2. 208 qualitative comments were analysed and categorised under 23 common themes, and mapped under existing or new metrics. These comments led to nine new areas of practice for metrics (environmental restraints, meaningful and purposeful activities, sexuality and relationship, family centred care, advocacy, transition planning, life stages and social inclusion, nutritional health, long-term conditions, managing personal finances) creating 35 existing and nine new metrics for round 2.

The 233 nurses participated in the round 1 were sent invitation for the round 2. A total of 218 nurses completed round 2 with a response rate of 93.5%. In round 2, 43 of the 44 metrics met the inclusion criteria with only sexuality and relationship (68.37%) scoring as “very important” by less than 70%. Following round 2, a working group meeting was convened between intellectual disability nursing members, academic, nurse leaders and clinical experts and the 43 metrics were re-formulated into 12 metrics. Seven of these new metrics had little or no supporting literature and required indicator development by the working group members before distribution in round 3 of the e-Delphi survey.

The set of 12 metrics with their 95 respective indicators were sent to participants in round 3. All ID nurses who initially expressed their willingness to participate in the study before the first round were able to participate in round 3. Since there were 6 nurses dropped out during the first two rounds, 395 nurses were sent invitation for the round 3, thus round 3 had 234 participants and a response rate of 59.2%. Ninety-three of the 95 indicators associated with the 12 metrics reached the 70% threshold and these were included. The two indicators which did not reach the 70% threshold were excluded and related to environment and safeguarding metrics. Also within round 3, nurses were given the opportunity to add comments and suggest other indicators. 88 qualitative comments were provided and following analysis the indicators were refined, merged, or separated where needed, resulting in 84 indicators going forwarded to round 4. The 234 nurses participated in the round 3 received the invitation link for round 4 which was conducted with participation of 177 nurses, thus had a response rate of 75.6%. All indicators distributed in round 4 of the e-Delphi Survey were rated above 70%. This resulted in 12 intellectual disability metrics and 84 associated indicators prior to consensus meeting.

To complete the process a face-to-face final consensus meeting was held in November 2017 with participation of key stakeholders. Each metric and respective indicator was discussed and voted on, with guidance provided to clarify ground rules in the consensus meeting. Some editing to wording was agreed and through discussion some indicators were collapsed prior to voting. This resulted in all 12 metrics and 79 of the 84 associated indicators reaching the 70% threshold to be included in the new set of intellectual disability nursing process quality care metrics and indicators (Table 3).

**Table 3:** Final set of Intellectual Disability Nursing Process Metrics and Indicators

Metrics	Indicators
1. Nursing Documentation	Nursing written records are legible, in permanent ink and signed
	Documented alterations/corrections are as per NMBI Guidance
	Personal information is stored securely with access only to relevant persons in order to protect the privacy and confidentially of the individual’s details
	Documented entries are dated and timed (24 h clock)
	Documented entries are in chronological order
	Documented abbreviations/grading systems are from a national or local approved list/system
	All student nurse documented entries are countersigned by the supervising nurse
2. Medicines Management	All medicinal products are stored in a locked cupboard/trolleys/or room
	Misuse of Drugs Act (MDA) are checked & signed at each shift changeover by registered nursing staff (member of day & night staff)
	Two signatures are entered in the MDA Drug Register for each administration of an MDA.
	The MDA cupboard is locked and keys are held by the designated nurse
	MDA drug keys are kept separate from other medication keys
	The person’s prescription documentation provides details of person’s legible name, unique identifier and photo ID
	The Allergy Status is clearly identifiable on the front page of the prescription chart
	Prescribed medicines not administered have an omission code entered and appropriate action taken
	The prescription start date is recorded
	The correct legible dose of drug is recorded with correct use of abbreviations
	The route and/or site of administration is recorded
	The frequency of medicines administration is as prescribed
	The minimum dose interval and/or 24 h maximum dose is specified for all PRN medicines
	The prescription has the prescriber’s signature (in ink) and Medical Council Number/Nursing and Midwifery Board of Ireland personal identification number
	Discontinued medicines are crossed off, dated and signed by person with prescriptive authority
	All medicines are reviewed in accordance with medication protocols
	A current Drug Formulary is available at the point of administration
	The generic name is used for each medicine unless the prescriber indicates a branded medicine and states “do not substitute”
	There is a support plan for self-administration of medication
	Self-administration of medicines is monitored for compliance and safety
3. Environment	Policies, Procedures, Protocols and Guidelines (PPPGs) are current and signed by each registered nurse
	There is evidence of an action plan based upon the most recent regulatory inspection
	Environmental and infection control audits have been conducted and relevant action plans are in place
4. Safeguarding	Safeguarding policies are reviewed and up to date
	Information is provided to the person regarding their rights (support to exercise their rights, advocacy, safeguarding/protection) in accessible formats
	Where there is evidence of a safeguarding concern there is documentation of registered nurses compliance with the safe guarding policy
	A personalised risk assessment has been carried out in consultation with the person and relevant persons (family, advocates and the multidisciplinary team) and evident in the nursing care plans
	A plan is in place on the person’s personal property, finances and possessions
	When assisting the person in the management of their finances, there is evidence that clear records are maintained, reconciled and subject to audit
5. Person centred communication	A communication assessment has been conducted and a plan is documented
	The person’s choice is obtained, respected and documented
	Communication strategies are identified in the persons care plan
	The person’s communication level and style are documented
	Non-verbal and atypical communication behavioural patterns are documented
	There is documented evidence of a multidisciplinary team approach
	Information provided is in an accessible format for the individual
	Where non-engagement occurs, this is noted in the persons care plan
6. Physical health assessments	A comprehensive health assessment has been conducted
	Known associated health risk factors are identified within the care plan
	A recognised assessment tool for persons with an intellectual disability has been used or appropriate tool adapted for specific areas e.g. pain, oral care, nutrition, hydration
	The person has been supported to engage in health screening
	The health care plan demonstrates a systematic approach to nursing care, management and interventions
	Physical health checks are conducted at least annually
	An individualised health passport has been developed in conjunction with the person
7. Mental health assessment	A nursing mental health assessment has been conducted and documented
	A diagnosis of mental health illness is documented
	The individuals care plan demonstrates the nursing care, management and interventions to support the person’s mental health and well-being
8. Risk assessment and management	There is evidence of positive proactive risk assessment and an action plan for identified risks within the persons care plan
	Appropriate referral and resulting consultations have occurred to address identified risks and are documented
	Incidents are documented within the care plan and escalated/reported as appropriate
	A risk re-assessment is conducted and documented
9. Nursing care plan	The personal plan is based on a model of care (Nursing Care Plan is based on an identified model of care)
	An assessment of need has been conducted and documented
	An individualised plan of care has been developed
	All documented nursing interventions are dated, timed and signed
	The care plan reflects the persons’ current health needs
	There is evidence of regular review of the care plan, dated, timed and signed
10. Person centred planning	A personal plan/assessment of all aspects of the person’s life has been conducted
	Actions/interventions are devised to support the person within their personal plan
	There is evidence of the person’s involvement in their Personal Plan
	The person’s level of need and preferences regarding the provision of intimate personal support are identified
	Self-advocacy/choices are recorded, respected and documented
	A transition plan exists across each life course stage
11. Positive behaviour support	An assessment of distress has been conducted
	A personal behavioural plan exists
	Proactive and reactive behavioural strategies are identified and evident
	There is evidence that positive behavioural support strategies are reviewed by the multidisciplinary team
12. End of life/palliative care	An end of life care plan is evident and documented
	The person has been supported to make end of life decisions and this process is evident within the personal care plan
	An ongoing assessment of changing health needs is evident and document
	A collaborative approach is in evident across services
	There is evidence of ongoing information sharing with the individual regarding their end of life

## Discussion

Although the grey literature identified a pre-existing set of ID nursing metrics developed in Ireland, in the literature review there was a lack of developed metrics in which all the traits of a metric (care process, standard and measurement) were present. In addition, the metrics that were identified from the literature review were developed from general nursing evidence rather than ID nursing. This can be partly explained by the fact that specific ID nursing degree programmes are only offered in Ireland and the UK and generally fall within mental health nursing in other countries. Consequently, metrics and indicators related to ID nursing are limited in the international literature and the process metrics that do exist are often derived from generic nursing processes. While such evidence may have clinical relevance and applicability there is difficulty in using generic nursing process as the specific needs of people with ID are often absent [34].

The development of nursing metrics from and within the specialist area of ID nursing is essential as people with ID are living longer [35]. They experience earlier mortality [36], and are more commonly diagnosed with comorbidities than people without ID [37, 38]. This disparity is acknowledged as being due to inequity as opposed to having a solely physiological basis [39]. Persons with ID admitted to acute, general settings may have their needs unrecognised and thus unmet. This is partly explained as general nurses who have no ID nursing experience report feeling underprepared, inadequate and struggle with communication barriers [40]. This is not surprising given that most nurses may not have cared for a person with ID during their undergraduate training or studied any theoretical or applied content regarding best practice when nursing a person with ID [34]. The development and translation of ID nursing metrics into other nursing care environments can assist in dealing with the health inequalities experienced by people with ID and facilitate health equity [41]. However, as many health care organisations at present are not equipped or do not have trained staff to work with people with ID [42–44] there is a need to address matters relating to service provision such as; metrics, continuous professional development and education.

Given the lack of research evidence underpinning ID nursing metrics and indicators, different forms of evidence including practice evidence and service user evidence were needed in guiding the development of the metrics and indicators within this project. The incorporation of the grey literature was essential in identifying aspects of nursing care process and areas of practice that warranted consideration based on views of practitioners, organisational policies and Irish and international regulatory authorities. Within this project, grey literature was sourced from ID services nationally and supplemented by hand searching to ensure a comprehensive search strategy and this literature provided valuable background to existing and potentially new metrics and should support implementation of the metrics given the practice evidence that was utilised and the involvement of practitioners within the project.

Given the absence of research evidence, ID nurses need to generate a robust accessible research base stressing the needs of people with ID and effective care strategies to meet those needs [45]. Such research should attempt to capture and illuminate the very heart and kernel of care in order to develop an evidence-based quality service [14]. This is further emphasised by the development of ID nursing over the years where, historically ID nurses worked in large facilities but with philosophical and funding changes many large institutions have closed and most people with ID are cared for by nurses working in community settings of various types such as; special education settings, small facilities, residential homes, or personal homes [46]. In addition, known that there is an ageing population and the health inequalities people with ID face across their lifespan there is a need for specialised nursing care [38, 47]. However, while ID nurses need to make their contributions visible [14] they often struggle to identify and explain what distinguishes them and sets them apart from other allied health professionals and the value nursing brings to the health care team [48, 49]. Thereby the production and use of ID nursing metrics will contribute and support quality care provision in a measurable manner.

ID nursing necessitates a wide range of specified skills so as to meet the varied health, advocacy, societal, behavioural needs of persons with ID. It is a complex role in that it is not a uniquely technical nursing role, but a unique relational role due to the restrictions in intellectual function and adaptive behaviour [50, 51]. Nurses who have developed specific skills to work successfully with people with ID are crucial in safeguarding the delivery of high quality healthcare in a diversity of settings with improved outcomes for health and well-being. Recent reports have emphasised unsatisfactorily high numbers of preventable deaths of people with ID that can be accredited to limitations in communication, diagnostic overshadowing, misdiagnosis, poor standards of care and the denial of rights [52–56]. There is a need for nursing metrics developed from and within the specialist area of ID nursing to support quality care provision as they become embedded in healthcare governance and management systems [57].

Within the study it was important to be aware of the quality of the metrics and indicators. To assist this process the authors used a framework to enable the consensus group to evaluate metrics and indicators against the four key attributes of; process focused, important, operational and feasible. This ensured that the metrics and indicators were considered as process focused and important to ID nursing practice and practitioners. Within the tool the third domain (operational) raised some concerns as not all of the metrics and indicators had reference standards and/or research evidence supporting them. However, they had a strong practice evidence base and support of the consensus group. This in turn impacted on the final appraisal trait of feasibility as the lack of indicators within the literature have an impact on implementation. However, as the indicators were developed within the Delphi process and by the work stream members’ one could argue that they will be feasible for practice implementation. Prior to implementation it is strongly recommended that metrics and indicators are piloted before complete usage to avoid unintentional and adverse consequences [58], thus pilot testing of these indicators is recommended and planned.

This study attempted to address this deficit by engaging key stakeholders nationally and this has resulted in a set of ID nursing process care metrics for Ireland. Given Ireland’s unique position in having ID nurse education and registration, the metrics developed are relevant, applicable and measurable for ID practice and can be adopted by other nursing care agencies across the world. This global applicability is important given the international quality and safety agenda and the necessity to support the rights of vulnerable people such as those with ID. In many international healthcare organisations the measurement of care is frequently suggested as a means to improve care outcomes and experiences [59]. In Ireland, Health Information and Quality Authority’s Regulations and Standards for Residential Services for Adults and Children with Disabilities (2013) [59] are set out under eight themes. The first four relate to the quality and safety of services provided and the remaining four relate to the workforce and their capacity and capability to provide services of appropriate quality and safety. The development of the set of ID process metrics in this study will assist in supporting quality and safety in ID services and support improved care experiences and outcomes. Developing a set of ID metrics will assist in having a clear standard and measurement of care delivery which is important given that variation in care delivery is well documented in both the literature and in reports and investigations of healthcare regulators. Quality of care is a complex concept and although it is central to modern healthcare policy [60, 61], it can be challenging to measure.

### Limitations

While this study produced a set of ID nursing metrics it also highlighted that the evidence underpinning the metrics is practice based rather than research evidence based. This may lead to applicability to practice but an evaluation of their implementation is needed to consider their use in Ireland and translation outside of Ireland. While the study aimed to represent nurses working in ID services nationally the only 4.9% of the total ID nurse population in Ireland participated across the Delphi phase. This reflects both non access to work emails in ID services and non-participation.

## Conclusion

The aim of the Nursing Quality Care Metrics project was to identify a final set of nursing quality care process metrics and associated indicators for ID to enable the provision of evidence of nursing’s contribution to safe, high quality patient care. Twelve nursing care process metrics and 79 indicators for ID were identified and having ID nursing metrics data available for use by front line staff and management levels creates the potential to measure fundamental nursing care processes and enable staff to make enhancements to minimise the risk to patients and their families. Nurses working in ID in Ireland are educated to degree level and are therefore well positioned to influence improvements and positive outcomes for clients in their care. However, the challenge to date has been the identification of the specific measures that are sensitive to the influence of nurses and having consensus that they are appropriate and relevant. The need for ID nursing specific metrics and indicators is evident as they utilise more specific teaching, social and emotional support focused approaches rather than a traditional medical model focused approach to care. Therefore, this project offers a unique contribution, in developing a set of national metrics and indicators for ID nursing through a process of involvement with key stakeholders and practitioners. The resulting metrics and indicators are pertinent to the explicit needs of people with ID and provide an opportunity to nurses internationally who support people with ID (either through general, mental health or developmental disability nursing services) to define and quantity the quality of care provided.

## Data Availability

The research data is available on request (dusezgin@gmail.com).

## Abbreviations

ASSIA: Applied Social Sciences Index and Abstracts
CCAT: Crowe Critical Appraisal Tool -
CDRS: Cochrane Database of Systematic Reviews
CENTRAL: Cochrane Central Register of Controlled Trials
CINAHL: Cumulative Index of Nursing and Allied Health Literature
CNM: Clinical Nurse Manager (with levels 1, 2, and 3)
DARE: Database of Abstract of Reviews of Effects
EMBASE: Excerpta Medical Database
ID: Intellectual Disability
MDA: Misuse of Drugs Act
NIDD: National Intellectual Disability Database
NMBI: Nursing and Midwifery Board of Ireland
NMPD: Nursing and Midwifery Practice Development
PPPGs: Policies, Procedures, Protocols and Guidelines
PRISMA: Preferred Reporting Items for Systematic Reviews and Meta-Analyses
PsyInfo: Psychological Information Database
Pubmed: Public Medline
UK: United Kingdom
